# GABA_B_ receptor encephalitis in a patient diagnosed with amyotrophic lateral sclerosis

**DOI:** 10.1186/s12883-019-1269-7

**Published:** 2019-03-14

**Authors:** Heike Schumacher, Thomas Meyer, Harald Prüss

**Affiliations:** 1 German Center for Neurodegenerative Diseases (DZNE) Berlin, Charitéplatz 1, 10117 Berlin, Germany; 2 Center for ALS and other motor neuron disorders, Charité – Universitätsmedizin Berlin, Augustenburger Platz 1, 13353 Berlin, Germany; 3 Department of Neurology and Experimental Neurology, Charité – Universitätsmedizin Berlin, Charitéplatz 1, 10117 Berlin, Germany

**Keywords:** Amyotrophic lateral sclerosis, GABA_B_ receptor encephalitis, Autoimmune encephalitis, Neuronal autoantibodies

## Abstract

**Background:**

In 2010 the spectrum of known antigens in autoimmune encephalitis has been expanded by GABA_B_ receptors. Until now over 80 patients with GABA_B_ receptor encephalitis have been described. We report the occurrence of GABA_B_ receptor antibodies in a patient with clinically diagnosed amyotrophic lateral sclerosis (ALS). GABA_B_ receptor antibodies have not been described previously in an ALS patient.

**Case presentation:**

A 75-year-old female patient presented with cerebellar ataxia, bulbar palsy and cognitive impairment. In the later course of disease signs for affection of the second motor neuron evolved and she was diagnosed with ALS. A post-mortem analysis of cerebrospinal fluid revealed high titers of GABA_B_ receptor antibodies.

**Conclusions:**

This case provides an idea of the natural course of GABA_B_ receptor encephalitis and demonstrates that antibody-mediated autoimmunity could be one of several pathways leading to the ALS phenotype. Furthermore this unique case stimulates the question whether neuronal antibodies might be more common in ALS than previously suspected.

**Electronic supplementary material:**

The online version of this article (10.1186/s12883-019-1269-7) contains supplementary material, which is available to authorized users.

## Background

The spectrum of antibody-mediated encephalitis has recently been expanded to GABA_B_ receptor (GABA_B_R) autoantibodies [1]. Most patients present with cognitive impairment and seizures [2], but also cerebellar ataxia and brainstem dysfunction [1–3] often with small cell lung cancer [4]. We describe a patient diagnosed with amyotrophic lateral sclerosis (ALS), in whom post-mortem analysis revealed high titers of GABA_B_R autoantibodies.

## Case presentation

**Fig. 1:**
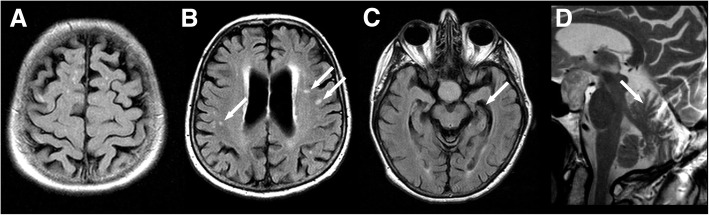
Cerebral MRI six months after symptom onset showed cortical atrophy (**a**), multiple, mainly periventricular white matter lesions (**b**), bilateral mesiotemporal atrophy (**c**) and cerebellar atrophy (**d**). The findings are not characteristic for ALS, but occur regularly in patients with GABA_B_ receptor encephalitis

In 2007, an otherwise healthy 75-year-old woman was admitted with progressive dysexecutive and behavioral syndrome, drowsiness, dysarthria and cerebellar signs, starting eight months earlier after severe bronchitis (Additional file 1). Neurologic examination revealed a pseudobulbar syndrome resulting in dysarthria and mild dysphagia, gait instability, bradydiadochokinesia, dysmetric finger-to-nose-test and saccadic eye movements. No fasciculations or tongue fibrillations occurred, eutrophic muscles had normal tone, pyramidal signs were negative, masseter reflex and motor-evoked potentials (MEPs) normal, EEG without epileptiform discharges, the patient had no fever or epileptic seizures. Electromyography showed generalized acute denervation and chronic neurogenic changes, nerve conduction studies showed motor-dominant neuropathy. MRI showed global atrophy and multiple white matter lesions (Fig. 1). Body CT with contrast and immunofixation was unremarkable. Cerebrospinal fluid (CSF) had elevated protein (90.1 mg/dl), normal cell count (3/μl) and no antibodies against CV2, Hu, Yo, Ri, Amphiphysin or acetylcholine receptors.

Five months later, pseudobulbar symptoms and cerebellar ataxia worsened. Fasciculations appeared at the trunk and all extremities showing an asymmetric but generalized spreading pattern. Bulbar symptoms, paresis and atrophy of limbs, shoulder girdle and hand muscles evolved. There was an increased muscle tone without hyperreflexia. MEPs remained physiological. The diagnosis of ALS was made (probable ALS according to revised El Escorial criteria) [5] and treatment with riluzole started. The patient had no family history of ALS. After three months, the patient received percutaneous endoscopic gastrostomy and non-invasive ventilatory support. She died 18 months after symptom onset.

Given some early symptoms not characteristic for ALS such as cerebellar signs, archived CSF and serum were tested for neuronal surface autoantibodies and revealed high titers (1:3200 in serum, 1:320 in CSF; cell-based assay, Euroimmun, Lübeck, Germany) of GABA_B_R IgG antibodies, while approximately 30 further antibodies (including LGI1, Caspr2, GABA_A_ and AMPA receptor) were negative.

## Discussion and conclusions

GABA_B_R antibodies have not been described previously in an ALS patient. Due to post-mortem identification of high-level GABA_B_R autoantibodies and the lack of immunotherapy, we cannot unambiguously determine whether the patient suffered from autoimmune encephalitis. Also no pathological test for the presence of TDP-43 could be performed. Given the similarity to published patients, we assume that our patient primarily developed GABA_B_R encephalitis followed by a secondary manifestation of clinical ALS symptoms. Indeed, the initial presentation encompassed symptoms not typical for ALS (including drowsiness and cerebellar symptoms), while dysphagia, dysarthria, hypomimia and cognitive impairment belong to the known symptoms in GABA_B_R encephalitis [1]. Elevated CSF protein and brain atrophy are also described [2, 4, 6]. A similar case of paraneoplastic GABA_B_R encephalitis with dysarthria, dysphagia, ataxia and respiratory failure was stabilized with methylprednisolone and plasma exchange [3].

As GABA_B_R are expressed in peripheral nerves [7] and throughout the CNS, particularly in the cerebellum [8], antibody-mediated interference with GABAergic transmission might have caused ataxia and axonal neuropathy [6]. As GABA_B_R antibodies were in the reported case not revealed before death and consequently no immunotherapy had been started, we cannot prove their contribution to disease. However, considering the high antibody titers and symptoms compatible with GABA_B_R encephalitis, they likely contributed to the severity of clinically diagnosed ALS, also given their pathogenic potential *in vitro* [9]. The findings give fresh impetus to the interpretation of autoimmunity as one of several etiologies of ALS, supported by findings of lymphocytic CNS infiltration [10] and IgG deposits [11].

The present case is remarkable for several reasons. First, the lack of immunotherapy potentially allows estimation of the natural course of GABA_B_R encephalitis which could develop into an ALS phenocopy. Second, screening for GABA_B_R antibodies seems useful in suspected ALS patients with cognitive impairment and ataxia. Third, it stimulates the question whether a systematic search for neuronal antibodies in ALS cohorts will reveal higher frequencies than hitherto suspected. It seems well possible that antibodies influence the ALS course. Potentially, antibody-mediated autoimmunity is one of several pathways leading to the ALS phenotype, comparable to HIV- or lymphoma-related ALS [12, 13].

## Additional file

Additional file 1: Timeline. The timeline gives an overview over the course of the disease of the patient and highlights diagnostic findings and therapeutic interventions. (PDF 25 kb)

## Abbreviations

ALS: Amyotrophic Lateral Sclerosis
CNS: Central Nervous System
CSF: Cerebrospinal Fluid
GABA_B_R: GABA_B_ receptor
MEP: motor-evoked potentials
