# Supplementary figures and images for: GABAB receptor encephalitis in a patient diagnosed with amyotrophic lateral sclerosis
Source: BMC Neurol. 2019 Mar 14;19:41. doi: 10.1186/s12883-019-1269-7 (PMC6416932; doi:10.1186/s12883-019-1269-7)

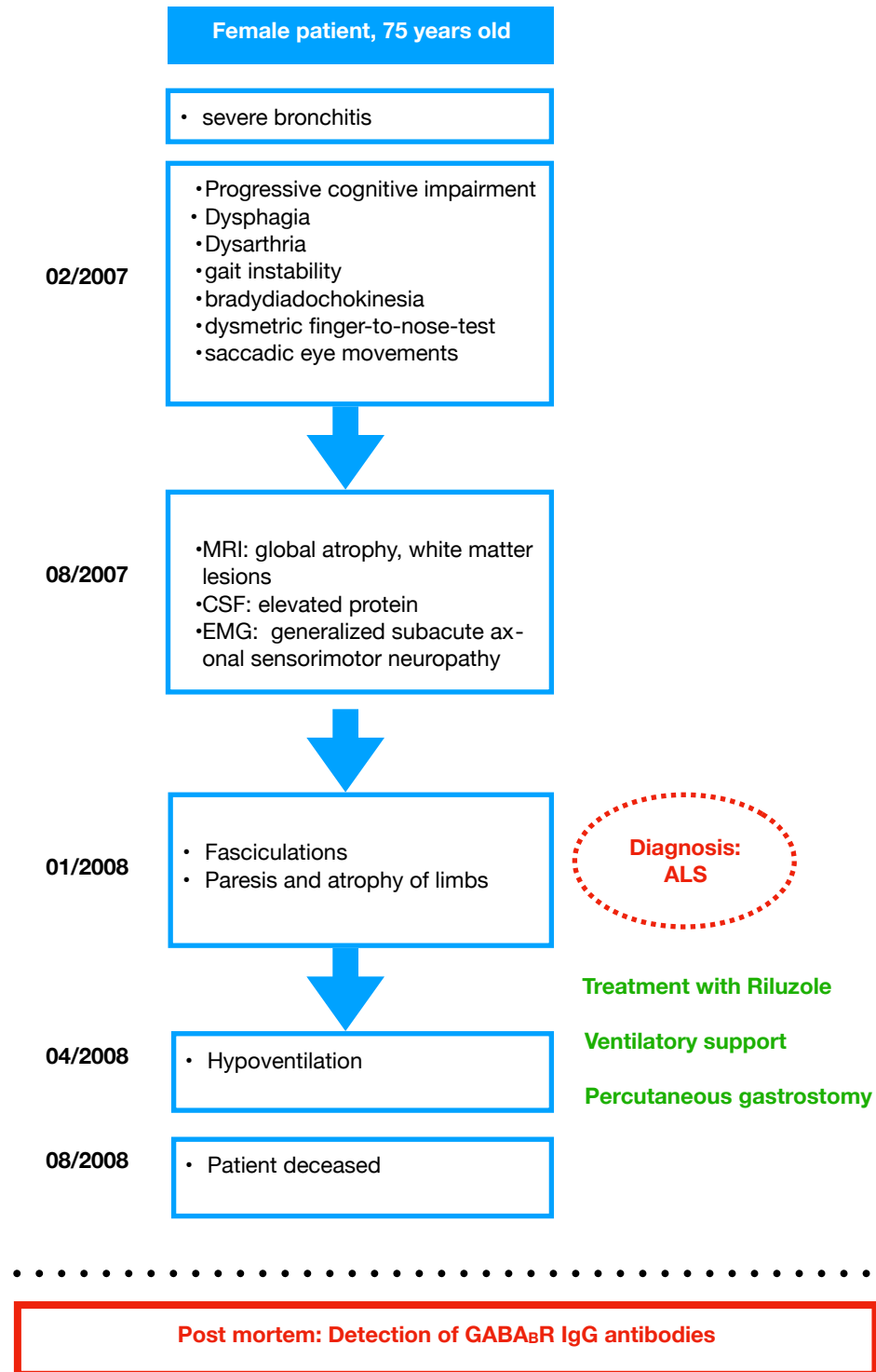

Supplement: Supplementary file 1 — Timeline. The timeline gives an overview over the course of the disease of the patient and highlights diagnostic findings and therapeutic interventions. (PDF 25 kb) [file 12883_2019_1269_MOESM1_ESM.pdf]
